# The complete mitogenome of *Tylomelania* sp. ‘Poso A’ (Caenogastropoda: Pachychilidae) from Lake Poso assembled from long-read sequencing data

**DOI:** 10.1007/s11033-026-11637-w

**Published:** 2026-03-06

**Authors:** Björn Stelbrink, Carola Greve, Charlotte Gerheim, Walter Salzburger, Thomas von Rintelen

**Affiliations:** 1 Department of Animal Ecology and Systematics, Justus Liebig University, Heinrich-Buff-Ring 26 (IFZ), 35392 Giessen, Germany; 2 Zoological Institute, Department of Environmental Sciences, University of Basel, Vesalgasse 1, Basel, 4051 Switzerland; 3 Senckenberg Research Institute and Natural History Museum Frankfurt/M, Senckenberganlage 25, 60325 Frankfurt am Main, Germany; 4 Museum für Naturkunde – Leibniz Institute for Evolution and Biodiversity Science, Invalidenstraße 43, 10115 Berlin, Germany

**Keywords:** Indonesia, Sulawesi, Freshwater gastropods, PacBio, Molecular phylogeny

## Abstract

**Background:**

Sulawesi’s ancient lakes represent biodiversity hotspots for various freshwater groups such as gastropods, shrimps, and crabs, as well as fishes. The less known Lake Poso in the northern part of the island is home to about 25 species belonging to the island-endemic freshwater gastropod genus *Tylomelania* within the family Pachychilidae. Here, we present the first complete mitogenome of a *Tylomelania* species from Lake Poso (*Tylomelania* sp. ‘Poso A’) assembled from long-read (PacBio) sequencing data.

**Methods and Results:**

The mitogenome is 16,540 bp long and thus only minimally shorter than the mitogenome of its congener from Lake Towuti, *T. sarasinorum* (16,632 bp). Based on 13 protein-coding and 2 rRNA genes we place the species within a recently published mtDNA-based phylogeny and show its close relationship to *T. carbo*.

**Conclusions:**

This is the first *Tylomelania* mitogenome to be generated using long-read sequencing data, and it is only the second complete mitogenome available for this species-rich radiation. Generating mitogenomes from such long-read data is only the first step towards creating the reference genome required for in-depth phylogenomic analyses.

## Introduction

The Indonesian island Sulawesi within the Malay Archipelago is a hotspot of biodiversity for various terrestrial, but also freshwater groups [1, 2]. The latter are particularly concentrated in the island’s ancient lakes and include morphologically and genetically diverse species flocks. These are monophyletic, species-rich, and endemic groups [3, 4], the most well-studied of which are gastropods, crabs, shrimps, and fish [5]. One of the most biodiverse and abundant freshwater invertebrate groups is the gastropod genus *Tylomelania*, which is endemic to the island and comprises about 50 mainly lacustrine species [5]. In recent years, evolutionary research has primarily focused on the well-studied Malili lake system. This freshwater feature was also the subject of an international deep-drilling endeavor, which revealed that Lake Towuti originated about 1 Ma [6]. In contrast, Lake Poso – located further north and not connected to the Malili lake system – is also very deep and species-rich (about 25 *Tylomelania* species, 3 gecarcinucid crabs, 8 *Caridina* shrimps, as well as 13 native and 17 non-native fishes; [5, 7, 8]), but its gastropod fauna is still insufficiently documented.

Much of the knowledge on species boundaries and phylogenetic relationships within Sulawesi’s freshwater species flocks is based on morphological and mitochondrial data (see, e.g. [5], for an overview). Despite the various advantages of mtDNA in taxonomy and systematics, several processes such as introgressive hybridization, incomplete lineage sorting, and horizontal gene transfer remain to be investigated (e.g. [9]). Moreover, young species groups, such as those inhabiting the ancient lakes of Sulawesi, may have evolved at a pace too quick to be detected for mitochondrial markers to resolve phylogenetically [5]. Therefore, genome-wide data is key to a better understanding of the relationships between these species and the role of introgression during the emergence of the different species flocks. For *Tylomelania*, genomic (short-read) data has been generated for the vast majority of species inhabiting the Malili lake system. However, this has not yet been exploited beyond the mitogenome level (see [10]).

**Fig. 1:**
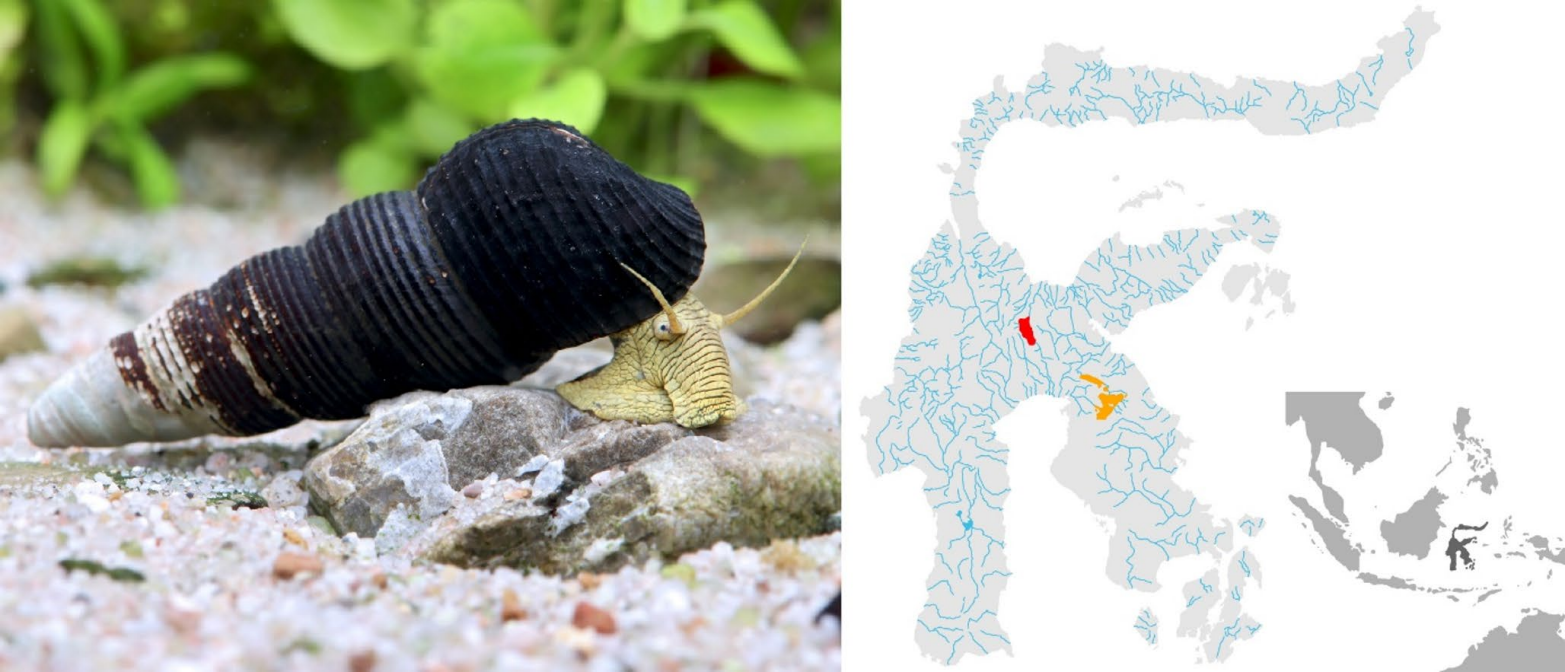
Example photo of *Tylomelania* sp. ‘Poso A’, taken in an aquarium (left panel). Photo by Chris Lukhaup (with permission). Middle panel shows Lake Poso (marked in red) and the Malili lake system (marked in orange) on Sulawesi including major drainages. Inset map (bottom-right) shows the island (marked in dark-grey) within the Indo-Australian Archipelago

As a first step towards gaining a more comprehensive insight into the evolutionary history of this genus, we here selected a specimen from a Lake Poso endemic as this can serve as a useful (genome) reference for subsequent phylogenomic studies. The strategy was to use a lacustrine species from outside the Malili lake system (see Fig. 1) as an outgroup, which exhibits similar features due to presumably undergoing similar adaptation processes in a similar environment (i.e., parallel evolution; see [11] for examples). Moreover, the reference species was selected to be genetically close enough to the remaining lake species facilitating mapping and SNP discovery in a genome-wide context. For the first time, we were using long-read sequencing on this genus in order to create an accurate and contiguous reference genome. To assess and make use of these long and high-quality reads, we first generated a mitogenome, which is presented here. This represents an important genomic contribution to the understudied Lake Poso fauna and will also help to shed more light on the extent of mitochondrial introgression within the genus.

## Materials and methods

A single individual of *Tylomelania* sp. ‘Poso A’ (see [11]; NCBI: txid246313) from an aquarium stock (Fig. 1) was selected for the genomic analysis to ensure that high-molecular-weight DNA can be extracted for long-read sequencing. The species is morphologically very distinct from the remaining species endemic to Lake Poso given its shell size and sculpture, and its body colouration (see [11]). The specimen and its DNA, which have been used here, are stored in the Lab Centre for Biodiversity Genomics of the Senckenberg Research Institute under voucher number TBG_4316.

Genomic DNA was extracted from foot tissue using a slightly modified CTAB protocol as described by [12], which is suitable for polysaccharide-rich tissue such as those from molluscs. Long reads were generated on a PacBio Sequel IIe at the Radboudumc Genome Technology Center’s Sequencing Core Facility in Nijmegen, the Netherlands. A total of 5,807,967 polymerase reads were yielded from the PacBio run. HiFi reads (read quality > 0.99) were obtained using pbccs 6.4.0 (github.com/PacificBiosciences/ccs), demultiplexed with lima 2.9.0 (github.com/PacificBiosciences/barcoding) and cleared from PCR duplicates using pbmarkdup 1.0.3 (github.com/PacificBiosciences/pbmarkdup). The final dataset consisted of 2,994,657 reads for further analyses.

HiFi reads were used to assemble the mitogenome of the *Tylomelania* sp. ‘Poso A’ *de novo*. The MitoHiFi 2.0 [13] pipeline was used to assemble the mitogenome *de novo* using the published *T. sarasinorum* mitogenome [14] as the source (i.e., reference for mapping) and to annotate the final contig using the implemented software MitoFinder [15]. Differences between the two complete *Tylomelania* mitogenomes were visualized and assessed in Geneious Prime 2025.2.2 (geneious.com).

To place *Tylomelania* sp. ‘Poso A’ within the *Tylomelania* Lake Poso clade, we made use of a recently published mitogenome dataset including 13 CDS and 2 rRNAs [10]. Single-locus datasets were concatenated using AMAS [16] and subjected to IQ-TREE 2.2.0 [17, 18] for phylogenetic analysis. We reduced the published dataset [10] by only including *T. carbo* (GenBank acc. no. for the 15 mitochondrial markers available at: ncbi.nlm.nih.gov/nuccore/?term=tylomelania+190828_1 & *+192946_1), *T. kuhli* (*+191710_1 & *+192321_1), *T. neritiformis* (*+190814_4 & *+190814_6) from Poso River (two individuals each) and the newly sequenced specimen of *Tylomelania* sp. ‘Poso A’ (GenBank acc. no. PX289796). We further included the two riverine species *Tylomelania* sp. ‘Beau’ (*+190750_4 & *+191027a_2) as well as *T. celebicola* (*+190529_5 & *+190532_4; also two individuals each), of which the individual *T. celebicola* (2714) was used as outgroup to root the tree (see [10]).

Two partition files were tested (15 partitions for the 13 protein-coding genes + 2 rRNAs vs. 41 partitions (codon partitioning for the protein-coding genes) with MFP+MERGE to identify best-fit substitution models and partition schemes. Nodal support was estimated using 1,000 ultrafast bootstrap (UFBoot) replicates and 10,000 replicates of the SH-like approximate likelihood ratio test (SH-aLRT; [19]). Nodes with > 95% for UFBoot and > 80% for SH-aLRT are considered well-supported [19].

## Results

**Fig. 2:**
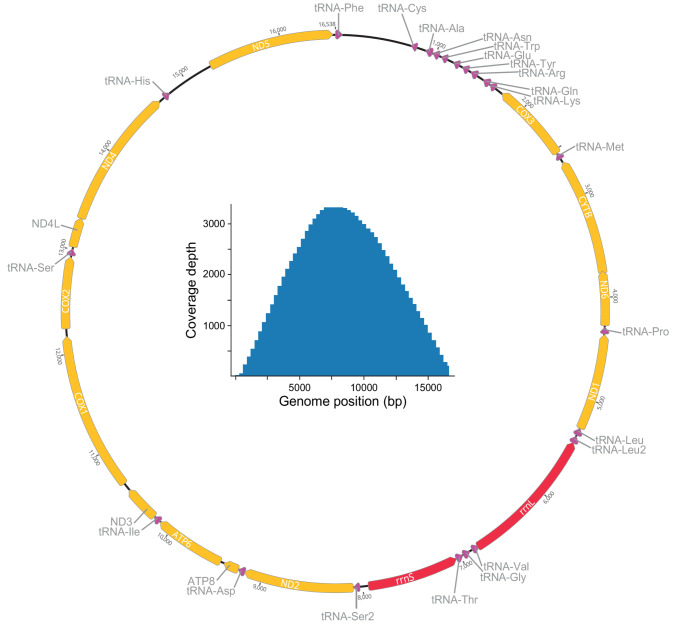
Annotated mitogenome of *Tylomelania* sp. ‘Poso A’ visualized in Geneious Prime 2025.2.1 (see Table 1 for more details on the features). Inset represents the output obtained from MitoHiFi showing the coverage depth along the assembled mitogenome (mean depth = 1,376.7, max depth = 3,384)

A total of 5,312 reads were mapped against the source with a mean and maximum coverage of 1,376.7 and 3,384, respectively (Fig. 2, inset). This is considerably higher than the mean coverage of 812.9 obtained from the short-read sequencing data analysed for the 78 *Tylomelania* species in [10]. More importantly, the present mitogenome is contiguous, whereas this only applied to 25 out of 78 mitogenomes in [10]. The assembled mitogenome is 16,540 bp long (Fig. 2) and thus only slightly shorter than that of *T. sarasinorum* (16,632 bp; [14]). Base composition was markedly biased towards a high A + T content (65.2%; A = 29.2%, T = 36.0%), whereas the G + C content was considerably low (34.8%; G = 18.6%, C = 16.2%). These ratios were very similar to those in *T. sarasinorum* (A + T = 65.2%, G + C = 34.9%). A single, longer repeat of 23 and 43 bp (TA motif) was identified within the D-loop/control region in the present and the *T. sarasinorum* mitogenome, respectively.

**Table 1:** Feature table of the mitogenome of *Tylomelania* sp. ‘Poso A’

Start (bp)	End (bp)	Type	Gene	Product
1	68	tRNA	*tRNA-Phe*	
809	747	tRNA	*tRNA-Cys*	
964	897	tRNA	*tRNA-Ala*	
1039	972	tRNA	*tRNA-Asn*	
1124	1059	tRNA	*tRNA-Trp*	
1257	1194	tRNA	*tRNA-Glu*	
1354	1290	tRNA	*tRNA-Tyr*	
1457	1388	tRNA	*tRNA-Arg*	
1594	1528	tRNA	*tRNA-Gln*	
1669	1602	tRNA	*tRNA-Lys*	
2533	1754	CDS	*cox3*	Cytochrome *c* oxidase subunit 3
2601	2536	tRNA	*tRNA-Met*	
3773	2634	CDS	*cob*	Cytochrome *b*
4278	3727	CDS	*nd6*	NADH dehydrogenase subunit 6
4346	4283	tRNA	*tRNA-Pro*	
5288	4356	CDS	*nd1*	NADH dehydrogenase subunit 1
5356	5289	tRNA	*tRNA-Leu*	
5438	5369	tRNA	*tRNA-Leu*	
6816	5432	rRNA	*16 S-rRNA*	
6868	6799	tRNA	*tRNA-Val*	
6968	6903	tRNA	*tRNA-Gly*	
7050	6979	tRNA	*tRNA-Thr*	
7948	7049	rRNA	*12 S-rRNA*	
8033	8099	tRNA	*tRNA-Ser*	
8100	9173	CDS	*nd2*	NADH dehydrogenase subunit 2
9172	9238	tRNA	*tRNA-Asp*	
9240	9410	CDS	*atp8*	ATP synthase F0 subunit 8
9432	10,127	CDS	*atp6*	ATP synthase F0 subunit 6
10,135	10,204	tRNA	*tRNA-Ile*	
10,205	10,558	CDS	*nd3*	NADH dehydrogenase subunit 3
10,635	12,167	CDS	*cox1*	cytochrome *c* oxidase subunit 1
12,238	12,927	CDS	*cox2*	cytochrome *c* oxidase subunit 2
12,940	13,006	tRNA	*tRNA-Ser*	
13,024	13,314	CDS	*nd4l*	NADH dehydrogenase subunit 4 L
13,308	14,675	CDS	*nd4*	NADH dehydrogenase subunit 4
14,731	14,798	tRNA	*tRNA-His*	
14,799	16,520	CDS	*nd5*	NADH dehydrogenase subunit 5

After comparing both mitogenomes, the final annotation of the present mitogenome was modified: (1) the range of 16 S-*rRNA* was extended from 5,432 to 6,814 bp to 5,432–6,816 bp to start at the same position as the one of *T. sarasinorum*; (2) the range of *atp8* was extended from 9,240 to 9,392 bp to 9,240–9,410 bp until a stop codon (TAA) was reached; (3) the range of *nd5* was extended from 15,274 to 16,518 bp to 14,799–16,520 bp until a fragment size was reached that was observed in other *Tylomelania* species [10] and that began with a starting codon (ATG); moreover, a ‘GA’ [coverage = 773] insert was added after 15,310 bp that was missing in the consensus sequence and thus caused the presence of multiple stop codons across the gene; and (4) the range of *atp8* was extended from 9240 to 9392 bp to 9240–9410 bp until a stop codon (TAA) was reached.

On the heavy strand, gene order was: *tRNA*^*Phe*^ (68 bp), *tRNA*^*Ser2*^ (67 bp), *nd2* (1,074 bp), *tRNA*^*Asp*^ (67 bp), *atp8* (171 bp), *atp6* (696 bp), *tRNA*^*Ile*^ (70 bp), *nd3* (354 bp), *cox1* (1,533 bp), *cox2* (690 bp), *tRNA*^*Ser1*^ (67 bp), *nd4l* (291 bp), *nd4* (1,368 bp), *tRNA*^*His*^ (68 bp), and *nd5* (1,206 bp). Gene arrangement on the light strand was: *tRNA*^*Cys*^ (63 bp), *tRNA*^*Ala*^ (68 bp), *tRNA*^*Asn*^ (68 bp), *tRNA*^*Trp*^ (66 bp), *tRNA*^*Glu*^ (64 bp), *tRNA*^*Tyr*^ (65 bp), *tRNA*^*Arg*^ (70 bp), *tRNA*^*Gln*^ (67 bp), *tRNA*^*Lys*^ (68 bp), *cox3* (780 bp), *tRNA*^*Met*^ (66 bp), *cob* (1,140 bp), *nd6* (552 bp), *tRNA*^*Pro*^ (64 bp), *nd1* (933 bp), *tRNA*^*Leu1*^ (68 bp), *tRNA*^*Leu2*^ (70 bp), 16 S-*rRNA* (1,385 bp), *tRNA*^*Val*^ (70 bp), *tRNA*^*Gly*^ (66 bp), *tRNA*^*Thr*^ (72 bp), and *12 S-rRNA* (900 bp). Two main overlaps were observed between *cob* and *nd6* (47 bp) as well as *nd4* and *nd4l* (7 bp). All PCGs started with an ATG codon and stopped with a TAA codon except for *nd1*, *nd2*, and *nd3* (TAG stop codon). The complete features of the mitogenome with the position of each gene are provided in Table 1.

**Fig. 3:**
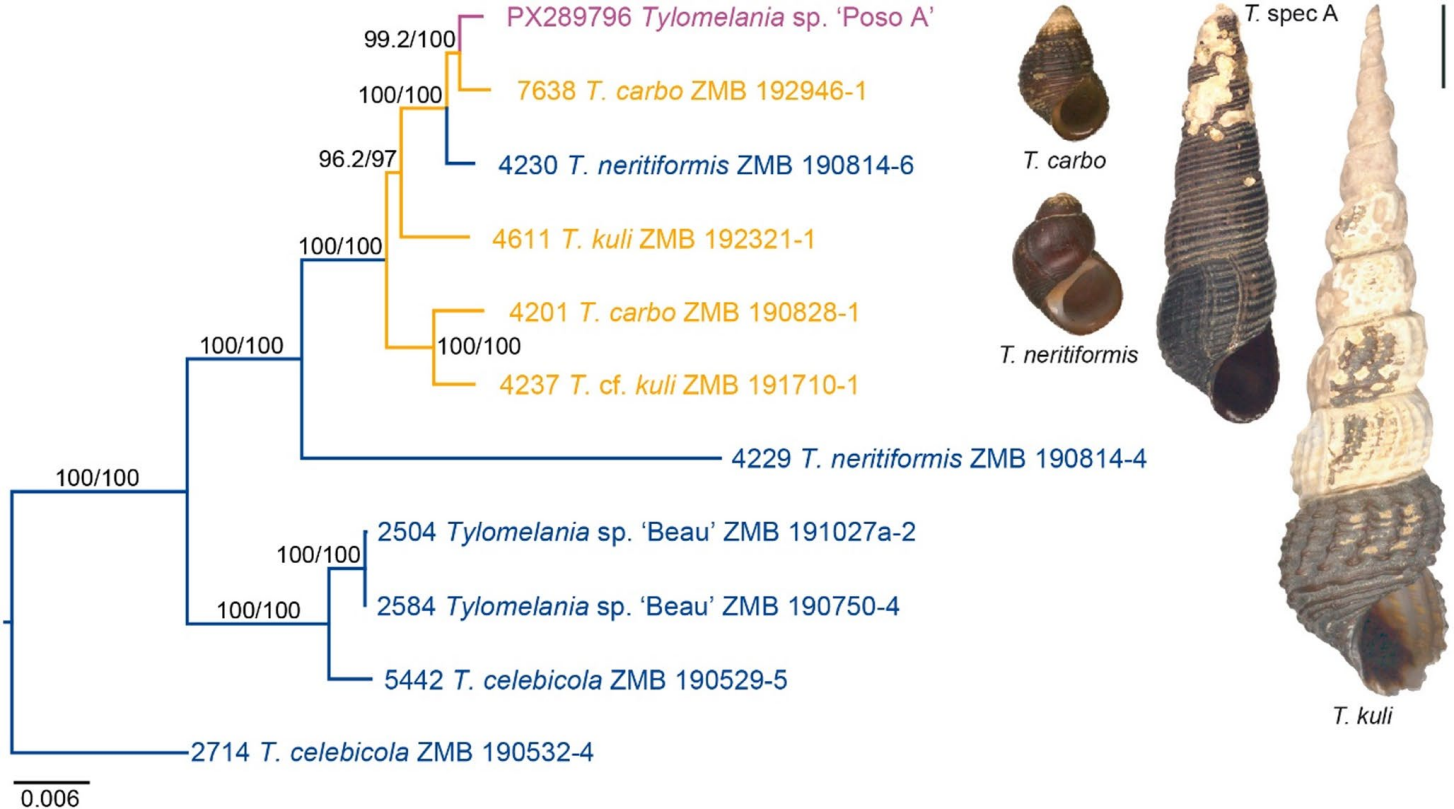
Phylogenetic relationships between *Tylomelania* from Lake Poso and the Poso River species (marked in orange) including *Tylomelania* sp. ‘Poso A’ (marked in pink) as well as selected riverine species (marked in dark-blue; see also [10] for a more comprehensive dataset). Numbers on branches indicate nodal support (left: SH-aLRT, right: UFBoot). Shells of Lake Poso species in apertural view are reproduced from [11] (scale bar = 10 mm)

MFP-MERGE favoured three and five partitions for the non-codon partitioning and the codon-partitioning scheme, respectively. As there was no difference between these two phylogenetic analyses in terms of topology and branch lengths, only the non-codon partitioning tree is shown (Fig. 3). Accordingly, *Tylomelania* sp. ‘Poso A’ clustered within the highly-supported clade P (Lake Poso; see also [10]) and is closely related to one of the two specimens of *T. carbo* from the lake’s east shore (Fig. 3). As in the previous study [10], the two individuals of each of these Lake Poso/Poso River species do not cluster together, but are scattered within the Poso clade.

## Discussion

Generating long-read sequences and a reliable mitogenome are the first steps towards a *Tylomelania* reference genome to be used in subsequent phylogenomic projects. Conducting (phylo)genomic studies on molluscs frequently poses challenges, for example due to the complexity of extracting high-purity, high-molecular-weight genomic DNA in the presence of high levels of mucopolysaccharides (e.g. [20–22]). Mollusc-specific CTAB-based isolation methods mitigate some of these issues and are widely used for traditional PCR and Sanger sequencing, but also for short-read sequencing approaches. Nevertheless, mucopolysaccharides may also affect long-read sequencing by interfering with the chemistry or blocking the pores used for sequencing [21]. We cannot evaluate this potential effect here, but the PacBio sequencing conducted appeared to be overall successful as reflected in the contiguous, highly covered, and well-annotated mitogenome. This contiguity is probably the main advantage of long reads for the mitogenome assembly, which could not be achieved for the majority of species analysed in [10], despite the high number of short reads and considerable mean coverage. Moreover, long reads help to better identify gene duplications, along with structural variants and tandem repeats in non-coding regions (e.g. [23]), which however were not observed here. A broader comparison is needed to validate possible structural differences and the accuracy of gene annotations. This should highlight the advantages and disadvantages of short-read and long-read approaches for mitogenome assembly.

In the present molecular phylogeny, the species from Lake Poso and the Poso River form a highly supported clade as in [10]. Discussing the closer relationship between *T. carbo* and *Tylomelania* sp. ‘Poso A’ remains difficult, as the former species has not yet been studied in a phylogenetic context, and the latter clustered within an unresolved clade [24]. Moreover, none of these species were recovered reciprocally monophyletic. Despite the limited dataset and the scattered distribution of the specimens across the lake, we assume that mitochondrial introgression could also play a significant role in Lake Poso as already discussed for the Malili lake system [10, 11].

## Conclusion

The non-monophyly of these morphospecies and several others within the Malili lakes (see also [10, 24]) highlights the need for other, non-mitochondrial, markers in future studies to postulate a more accurate species tree hypothesis (see also discussion in [10]). 

Such future phylogenomic studies will probably become increasingly collection-based given the threats to these fascinating ancient lakes. Habitat loss, increasing pollution, and particularly the introduction of the invasive fish species – such as the ‘flowerhorn’ cichlid (a man-made hybrid complex; see [25] for details) – to Lake Matano around 2005 [25–27] caused a decline in endemic biodiversity in recent years. In Lake Poso, where the present *Tylomelania* species occurs naturally, the situation is probably not much better. The fact that the fish fauna today consists mainly of non-native species that may feed on the endemic fauna should be a cause for concern [8]. Initiatives to protect these species are thus urgently needed.

## Data Availability

The mitogenome is deposited at NCBI’s GenBank under accession number PX289796. GenBank accession numbers for the 15 mitochondrial markers of the remaining species analysed can be found at: ncbi.nlm.nih.gov/nuccore/?term=tylomelania+190828\_1,*+192946\_1,*+191710\_1,*+192321\_1,*+190814\_4,*+190814\_6,*+190750\_4,*+191027a\_2, *+190529\_5, and *+190532\_4.
